# The Late Effects of Cancer Treatment on Female Fertility and the Current Status of Fertility Preservation—A Narrative Review

**DOI:** 10.3390/life13051195

**Published:** 2023-05-17

**Authors:** Kenny A. Rodriguez-Wallberg, Yanyu Jiang, Tobias Lekberg, Hanna P. Nilsson

**Affiliations:** 1 Department of Oncology-Pathology, Laboratory of translational Fertility Preservation, Karolinska Institutet, SE-17177 Stockholm, Sweden; yanyu.jiang@ki.se (Y.J.); tobias.lekberg@ki.se (T.L.); hanna.nilsson@ki.se (H.P.N.); 2 Department of Reproductive Medicine, Division of Gynecology and Reproduction, Karolinska University Hospital, SE-17177 Stockholm, Sweden; 3 Breast, Endocrine tumors and Sarcoma Cancer Theme, Karolinska University Hospital, SE-17177 Stockholm, Sweden

**Keywords:** fertility preservation, gonadotoxicity, oncofertility, cryopreservation, premature ovarian insufficiency

## Abstract

Fertility counseling should be offered to all individuals of young reproductive age early in the patient’s trajectory following a cancer diagnosis. Systemic cancer treatment and radiotherapy often have an inherent gonadotoxic effect with the potential to induce permanent infertility and premature ovarian failure. For the best chances to preserve a patient’s fertility potential and to improve future quality of life, fertility preservation methods should be applied before cancer treatment initiation, thus multidisciplinary team-work and timely referral to reproductive medicine centers specialized in fertility preservation is recommended. We aim to review the current clinical possibilities for fertility preservation and summarize how infertility, as a late effect of gonadotoxic treatment, affects the growing population of young female cancer survivors.

## 1. Introduction

The incidence of cancer in 15–39-year-old adolescents and young adults (AYA) is increasing, and in 2019, the global rate was 52.3 cases per 100,000 [1]. In AYA, carcinomas (approx. 30%) are most frequent, followed by lymphomas, melanomas, and tumors of the central nervous system, but the distribution changes with increasing age, and in older pre-menopausal women, carcinomas, specifically breast cancer (BC) (>40%), is followed by melanoma, cervix, and central nervous system tumors [1]. However, most cancers are also increasingly treatable, as shown by a continuously improved five-year survival rate in both children and AYA [1,2,3]. Together, these trends contribute to a growing group of cancer survivors of fertile age. However, many young cancer survivors will develop late effects of their treatment, causing a lifelong impact on mental and physical health, negatively affecting the quality of life [4,5]. Among common side effects of chemotherapy or radiotherapy are premature ovarian insufficiency (POI) and the risk of infertility. There are clinically established measures to preserve fertility both before and after cancer treatment, and for the best effect, these options should be evaluated as early as possible after diagnosis [6,7]. The goal of this review is to highlight current methods for fertility preservation (FP) and the clinical importance of gonadotoxicity as a secondary sequela of cancer therapy. Limitations of this manuscript are related to design (use of a narrative, rather than systematic, review) and scope (e.g., not all sequelae of cancer therapy have been included in this review, nor is each topic reviewed in depth). This review is written from a clinical point of view with a focus on recent evidence contributing to the evolvement of the available FP measures.

### Indications for Fertility Preservation

The European Society of Human Reproduction and Embryology, ESHRE, published updated guidelines for fertility preservation in 2020, including clinical recommendations for female FP and patient information brochures. This information is available in open access [7], and the work has also been summarized in a short article [8]. Similar guidelines have been published by other societies around the world [9,10,11].

**Figure 1:**
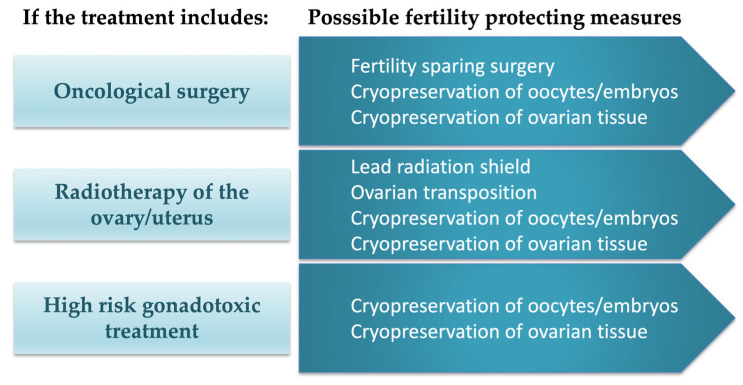
Fertility preservation measures. Available options sorted on proposed treatment. Adapted from Rodriguez-Wallberg KA, Oktay K, 2014 [14].

The most common indication for FP measures is planned cytostatic drug treatment, but oncological surgery and radiotherapy over the reproductive organs can also negatively impact future fertility potential, especially if the gonads or the uterus are located directly in the radiation field (Figure 1) [6,12,13,14]. In women who undergo hysterectomy or where radiotherapy irreversibly damages the uterus and the endometrium, FP measures can be applied through the use of the patient’s own gametes or embryos in later gestational surrogacy, which is allowed in several countries. However, in countries with prohibitive legislation regarding surrogacy, there are currently no clinically established means to assist in achieving pregnancy for these patient groups [15,16,17]. 

Among the cytostatic drug treatments, it is mostly alkylating agents in high doses that are associated with gonadal damage and infertility. The induced damage often leads to a severe reduction in the number of primordial follicles (PMF) that constitute the ovarian reserve. Table 1 summarizes chemotherapy and its association with the known risk of infertility [6,12,18,19].

**Figure 2:**
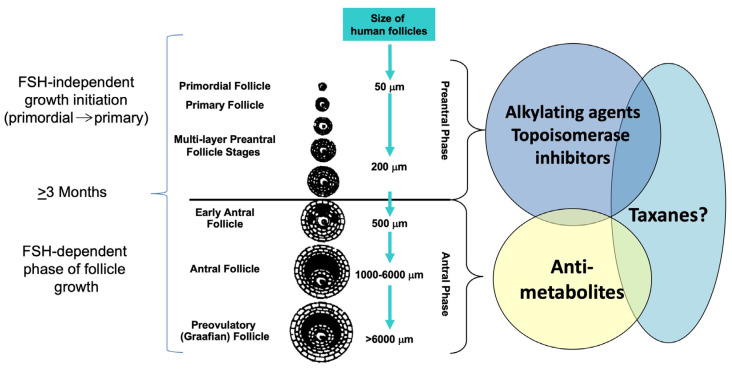
Impact of breast cancer chemotherapy on different stages of follicle growth. All chemotherapeutics are capable of damaging developing follicles, as their granulosa cells are proliferating and will cause at least temporary amenorrhea. However, if new follicles can develop from an undamaged primordial follicle population, this amenorrhea is only temporary for non-DNA-damaging agents. Alkylating agents and topoisomerase inhibitors directly damage DNA and result in a permanently shortened reproductive life span. Anti-metabolites such as methotrexate appear to only cause transient amenorrhea. The impact of taxanes on the primordial follicle population is inconclusive. Abbreviation: FSH, follicle-stimulating hormone. Figure reprinted from Rodriguez-Wallberg and Oktay, 2012 [24], with permission.

While clinical studies can provide convincing evidence of ovarian damage after chemotherapy, it is often difficult to distinguish the gonadotoxic effect of a single cytostatic drug in clinical trials as they are frequently used in combination therapies [18,20]. Figure 2 shows cytostatic agents used in breast cancer treatment and their impact on specific follicle stages. Alkylating agents, such as cyclophosphamide, and topoisomerase inhibitors, such as doxorubicin, have been shown to directly affect the primordial follicle reserve, which results in the shortening of the reproductive life span. In molecular studies, it has been shown that these agents induce apoptotic cell death in the nonproliferating population of primordial follicles via induction of DNA double-strand breaks [19,21]. Antimetabolites, such as methotrexate, appear to only affect the developing follicle population and, hence, cause transient amenorrhea without altering the ovarian reserve [22]. The impact of taxanes on the primordial follicle population has shown inconclusive results in clinical observations [18]. The recently published results from the NeoALLTO study indicate that taxanes could have a direct negative impact on fertility, as weekly addition of paclitaxel to the non-gonadotoxic anti-HER2 treatment in women with HER2-positive early BC resulted in a significant decline of Anti-Müllerian hormone (AMH) during the treatment. No data is yet available on AMH recovery [23].

## 2. Protecting the Ovarian Reserve In Vivo

Chemotherapy damage to the ovary can be inflicted through induced loss of primordial follicles. The impact of chemotherapy on human ovarian function is based on the presence of a finite PMF pool formed already in utero [25]. PMF growth, maturation, and atresia are triggered throughout the reproductive lifespan, and the continuous depletion of the follicular reserve ends in menopause. Folliculogenesis is strictly regulated, and most growing follicles are destined to become atretic at some stage during maturation, as only one oocyte will be fully matured in each ovulatory cycle [26,27]. The high proliferation rate of the granulosa cells surrounding growing follicles during the FSH-dependent phase of folliculogenesis makes them a target of many chemotherapy agents. The oocyte itself is also a sensitive target for DNA-damaging chemotherapy agents, as it needs to maintain its chromosomal and DNA integrity from formation until ovulation [28]. The two main mechanistic hypotheses of chemotherapy-induced follicular damage include either accelerated activation of primordial follicles and a fast burn-out of the follicular reserve or direct follicular damage through apoptosis, follicle atresia, inflammation, and vascular and stromal tissue damage [19,28]. Drugs that strictly act on the growing follicle often cause temporary amenorrhea, while drugs acting on the dormant follicle pool has the potential to affect long-term fertility [29].

### GnRH Analogues

GnRH agonists (GnRHa) are empirically used clinically off-label to protect ovarian function during cytotoxic treatment. The efficacy of the treatment is still under debate and subject to a large number of randomized controlled trials, both completed and ongoing, particularly in women with breast cancer. The available results are conflicting and have been widely analyzed and discussed [30]. The American Society of Clinical Oncology’s (ASCO) expert panel does not recommend the use of GnRHa as a method for fertility preservation in its update of fertility preservation guidelines for patients with cancer published in 2018 [9]. The use of GnRHa does show potential in reducing the prevalence of POI in women treated for BC [31,32], but it also appears that it does not protect the ovarian reserve, as reflected by lack of AMH recovery [32,33,34]. The efficacy of GnRHa in other cancers is unclear, and the largest trial, in women with lymphoma, showed no apparent benefit [35]. Additionally, the benefits in terms of avoiding the consequences of POI, for example, osteoporosis, remain to be evaluated.

## 3. Fertility Preservation as an Acute Measure

In several European countries, fertility preservation, including surgical methods or methods involving cryopreservation and assisted reproduction are state funded if clinically indicated, and the use is regulated according to the general rules for assisted reproduction, including an age restriction for treatment [36]. While the importance of FP is increasingly acknowledged, results from the recent Italian PREFER study showed that as many as 11.7% of all women eligible for FP measures denied the procedure, mostly out of fear of delayed cancer treatment [37]. Our research group in Sweden has compiled data from Karolinska University Hospital’s program for FP in several publications and has reported on the efficacy and safety of these procedures [38,39,40,41]. Additional studies encompassing Swedish nationwide data also indicate that the procedures for fertility preservation, including hormonal stimulation in women with BC, are effective and safe [42,43,44]. A recent meta-analysis on FP in BC patients supports these results and indicates that fertility preservation poses no increased risk for cancer recurrence; in addition, it observes an average of only 6 days delay in chemotherapy initiation after FP [45].

### 3.1. Cryopreservation of Oocytes and Embryos

In order to freeze a large number of oocytes or embryos during FP, the ovary is stimulated hormonally with gonadotropins, similar to regular in vitro fertilization (IVF) treatments. If the time for stimulation is limited, which is more rule than exception after a cancer diagnosis, stimulation is usually planned to use a short protocol [46]. This protocol uses GnRH antagonists in parallel with gonadotropins and can be initiated with “random start stimulation” at any time during the menstrual cycle. The method has been validated in large prospective studies and has shown better or equivalent results in terms of the number of eggs obtained, as the regular short protocol with stimulation starts on cycle day 1 [42,47]. If fertility is maintained, FP measures can also be applied after cancer treatment. To assess remaining fertility potential, a patient can be monitored for the level of AMH, which is a currently used clinical indicator of the female ovarian reserve [48]. Additional information can be obtained through transvaginal ultrasound, where the ovarian reserve is estimated by directly counting the antral follicles [43,49]. Pregnancy and live birth rates achieved per transferred embryo are similar in infertile patients and fertility preservation patients [50]. In recent studies, women with BC who have undergone hormonal stimulation to preserve frozen eggs or embryos have shown no increased risk of recurrence or death during follow up compared with women with similar cancers that did not undergo fertility preservation [38,42,43].

### 3.2. Hormone-Sensitive Breast Cancer

In women with breast cancer, it is important to take into consideration whether the tumor is hormone sensitive or not, as gonadotropin stimulation treatments, with the subsequent increase in endogenous estrogen production, have been considered unsafe. However, a recent meta-analysis showed no increase in cancer recurrence after controlled ovarian stimulation in patients with hormone-receptor-positive breast cancer [45]. To reduce the estrogen surge, an adapted stimulation protocol, including tamoxifen or aromatase inhibitors, has been recommended and is used with good results at several centers around the world [51,52,53]. In controlled studies, aromatase inhibitors alongside gonadotropins have been found more effective than tamoxifen [51]. In a commonly used regimen, a fixed dose of letrozole is used in parallel with FSH from the start of the cycle. Ovulation trigger with GnRH agonist is preferred because the endogenous estrogen production will be suppressed both during the stimulation and after the egg aspiration, which enables the patient to start cytostatic treatment on schedule [53]. Some studies have suggested a lower oocyte maturation rate when using the random start protocol in combination with letrozole [45], but this could not be confirmed in the results from a large prospective study [42] nor by a recent meta-analysis, where no negative effects either on oocyte maturation or on other efficacy and safety outcomes were observed [54].

When diagnosed with hormone-sensitive breast cancer, patients will often be recommended to complete several years of adjuvant endocrine treatment to suppress estrogen secretion or to block the estrogen receptors in the breast tissue. The most common drugs used include tamoxifen, GnRH analogs, and aromatase inhibitors, such as letrozole, exemestane, and anastrozole, used alone or in combination [55]. The large ATLAS study indicates that 10 years of adjuvant tamoxifen treatment is better than the previously recommended 5 years of treatment in reducing the risk of recurrence [56]. In many cases, at the end of long-term adjuvant endocrine treatment, a women’s residual fertility will have diminished due to natural ovarian aging, also when the woman has not received chemotherapy treatment [6,24]. Adjuvant endocrine treatment generally has low compliance, and an increasing number of women requesting to pause their adjuvant treatment in order to become pregnant has been reported [57]. The early results from the POSITIVE trial, which reports on women attempting pregnancy after interrupting endocrine treatment, found that the three-year cancer recurrence rate was not affected by up to a two-year break from adjuvant treatment [58]. Long-term follow up of the study is still ongoing.

### 3.3. The BRCA Mutation

The development of breast cancer at a young age indicates the need for screening for genetic cancer syndromes, such as those associated with mutations in the BRCA genes. Mutations in BRCA1 and BRCA2 are characterized by a hereditary increase in risk for both female and male BC, ovarian cancer (including fallopian tube and primary peritoneal cancers), prostate cancer, pancreatic cancer, and melanoma [24,59]. The BRCA mutation also affects the reproductive potential, as indicated by a reduced ovarian reserve, higher risk of POI, and decreased AMH [60,61,62,63]. Women with BRCA are often recommended prophylactic bilateral oophorectomy before the age of 40 to reduce the risk for ovarian cancers [63]. 

### 3.4. Cryopreservation of Ovarian Tissue for Later Re-Transplantation

An alternative method for fertility preservation is the retrieval, or biopsy, of the ovary to cryopreserve the cortical ovarian tissue where follicles are present [64]. In several fertility preservation programs, unilateral oophorectomy is currently applied [39,65]. Ovarian tissue cryopreservation (OTC) is the only FP measure currently available to prepubertal girls [66,67]. To regain fertility, the pieces of ovarian tissue are thawed and retransplanted into the body, where the follicles can mature in vivo. Re-transplantation can also be performed to regain endocrine function, in which case the transplant does not need to be orthotopic [68]. OTC is no longer regarded as experimental, but as innovative, according to ESHRE’s latest guidelines [7,8]. The recommendations still emphasize that clinically established methods for fertility preservation should be applied as a first option. Therefore, freezing of ovarian tissue is generally used in the absence of time for ovarian stimulation, in complicated cases with a contraindication to undergo either hormonal treatment or transvaginal egg retrieval, and in very young patients and pre-pubertal girls [67,69]. The ovarian tissue can advantageously be retrieved through a minimally invasive laparoscopic procedure that can be planned within just a few days [40]. 

The efficacy of the method is reduced by the detrimental effects caused via cryopreservation as well as ischemia-reperfusion injury at re-transplantation, but globally, the number of children born after successful re-transplantation is increasing [39,70,71,72]. The success rate of ovarian tissue freezing is dependent on a good ovarian reserve, and re-transplantation has been more effective in young women and girls. The international recommendation is to limit this fertility preservation measure to women younger than 35 years (Edinburgh criteria, published in 1996 and validated in 2014) [73]. Currently, the live birth rate after re-transplantation of ovarian tissue is estimated to be around 30%, but the usage rate is low [74,75]. The return rate is affected by a multitude of factors; the most common include a limited desire for pregnancy, spontaneous pregnancies, financial restrictions, ART age regulations, cancer relapse or death of the patient, fear of disease recurrence, and presence or absence of a partner [74]. 

Among BRCA carriers, the increased risk of malignancy also affects the risk involved in re-transplanting cryopreserved tissue, and current recommendations suggest removing the tissue once pregnancy has been achieved [7]. Additionally, in patients with systemic hematological disease, at the time of cryopreservation or tumors metastatic to the ovary, re-transplantation of cryopreserved gonadal tissue poses a risk of reintroducing malignancy. Currently, re-transplantation is only recommended when the risk of reseeding malignancy is regarded as unlikely, such as in cases when the tissue was retrieved following chemotherapy treatment that achieved complete remission prior to hematological stem cell transplantation [76].

### 3.5. In Vitro Maturation

Culturing small antral follicles from the germinal vesicle stage (GV) or metaphase I stage to maturity for 1–2 days before fertilization is currently regarded as innovative, while ex vivo maturation of immature follicles from cortical tissue is still experimental [7]. In vitro maturation is most commonly used for FP when there is a contraindication for hormonal stimulation, such as hormonal sensitivity or lack of time before gonadotoxic treatment [77]. As clinical studies have observed differences in embryo development during IVM, possibly with a negative effect on pregnancy and live birth rate [78,79,80], this has discouraged widespread clinical use. Additionally, while long-term follow up of the children born after IVM has, so far, not indicated detrimental effects with regard to health outcomes [81,82], concerns about safety remain. As shown in women with hematological cancers, IVM is a viable option for urgent fertility preservation [83], and the treatment has the promise to improve the mature oocyte yield and enable mature oocytes in cases where stimulation is discounseled.

## 4. Quality of Life and Late Effects

Infertility and its consequences have been shown to greatly impact the perceived quality of life after treatment completion, independent of where it is measured in the world, and is second only to concerns about disease recurrence [84,85,86]. The impact on the body’s natural hormone regulation and psychological side effects such as impaired self-image, depression, and unstable relationships can affect the will, ability, and possibility of reproduction [87,88]. In BC patients, family formation is delayed, and concerns about future infertility affect the choice of cancer treatment [89,90].

### 4.1. Premature Ovarian Insufficiency

After gonadotoxic treatments, there is a significant risk that fertility will be reduced even if menstruation returns. It is most common for menstruation to return within 3–4 months from the end of treatment, but it might take up to 2 years. Studies have shown that standard cytostatic treatment, with alkylating agents, leaves the woman with an ovarian reserve equivalent to an individual 10 years older [25,58,91], and after complementary radiotherapy, the effect is further enhanced [92]. If the ovarian reserve is completely depleted, menstruation does not return, and the woman becomes infertile; this is dependent on the intensity of treatment and also on age and the ovarian reserve at the time of cancer treatment. POI is more common if the women are older than 30 years of age at the time of treatment, but also, very young women can develop post-menopausal FSH levels [93]. The effects of going into premature menopause include not only infertility but also a higher risk of osteoporosis, uneven temperature regulation, muscle and joint pain, fragile mucous membranes, and mood swings. It is common to offer hormone treatments with estrogen and progestin to women in the event of amenorrhea or at suspicion of POI [94]. In young girls, induction of puberty can also be achieved via hormonal substitution, and the treatment should be continued to reduce the risk of osteoporosis [94,95], dementia [96,97], cardiovascular diseases, and premature death [98,99], all of which increase with POI at an older age. Treatment with bisphosphonates for secondary osteoporosis has been shown to have beneficial effects, including reduced fracture incidence, less cancer recurrence in bone tissue, and lower mortality in women treated for breast cancer [100].

### 4.2. Sexual Function

After cancer treatment, patients have both late side effects and trauma to process. Many experiences a lingering feeling of exhaustion. Both physical and psychological sexual function can be impaired, which also affects the quality of life negatively. Physical problems after treatment are often but not always linked to further sexual dysfunction of a psychological, cultural, or interpersonal nature [101]. Studies estimate that over half of cancer patients suffer from sexual problems after treatment, but only a small percentage actively seek help [101,102].

Radiotherapy of the abdomen and pelvis can lead to infertility, but also to additional side effects such as dry mucous membranes, bleeding after intercourse, and pain. Radiation to the brain can lead to disturbed hormonal levels and infertility but also to cognitive impairments that can make establishing and maintaining social relations more difficult [103]. Cytostatic treatment can lead to infertility and reduced estrogen production. Baseline results from an ongoing study on breast cancer reported that women treated with chemotherapy suffer greater physical and sexual difficulties [90]. All types of treatment can also cause neuropathy, where physical sensation is negatively affected. For women who have undergone allogeneic stem cell transplantation, Graft-versus-Host Disease (GvHD) is also a possible side effect. GvHD has been documented up to 8 years after transplantation and often occurs in the vagina with ulcers, adhesions, and pain from the mucous membranes as a result [104]. In general, women whose ovarian function has completely ceased have more sexual problems than women who continue to menstruate [105].

### 4.3. Psychological Effects

Infertility has far-reaching effects on mental health, emotional life, and social ability, where infertile women show a higher degree of anxiety and a higher proportion of psychiatric diagnoses [106,107]. Studies show that infertility mainly affects couples in four areas: psychological well-being [108], sexual relations [109], marital relations [109,110], and quality of life [110,111]. Even though many infertile couples nowadays have good chances of having children via IVF, this treatment also increases stress, hormonal impact, and tensions in the relationship [112,113,114].

In patients where fertility is preserved, cancer treatment can still be linked to reduced sexuality [115]. For the vast majority of women who have undergone cancer treatment, sexual dysfunction does not have a direct impact on fertility as they are postmenopausal at diagnosis. However, for all women, physiological changes can have direct effects on their psyche and body image. For example, after surgery, the size of the scarring is directly correlated to a negative self-image [116].

### 4.4. Pregnancy after Cancer

Studies indicate that hormonal stimulation treatments for fertility preservation aiming at oocyte or embryo cryopreservation do not increase the risk of cancer recurrence, but the risk of recurrence is still the biggest concern of cancer patients and might also influence decisions on future parenthood [40,42,117]. There is currently no data to indicate that it is dangerous to become pregnant and have children after being treated for cancer. A meta-analysis from 2022 even showed a slight reduction in recurrence rate after assisted reproductive techniques (ART) treatments compared to patients not exposed to ART for fertility preservation [45]. Another meta-analysis from 2021 showed similar pregnancy outcomes in breast cancer survivors compared to the general population. Furthermore, pregnancy prognosis after BC was not affected by tumor characteristics, previous treatment, the timing of pregnancy after BC, and BRCA status [118]. In a Swedish cohort of women with previous cancer treatment resulting in iatrogenic infertility, pregnancies and deliveries achieved via assisted reproduction with egg donation also indicate a good obstetric and perinatal outcome [119]. Only a slightly increased risk of gestational hypertension and prematurity was observed in women with previous cancer compared to the women who underwent similar assisted reproductive treatment with egg donation but had no history of cancer. However, cancer survivors are less likely to get pregnant [119]. A sibling study from 2016 showed that cancer patients undergoing cytostatic treatment run a 13% higher risk of not becoming pregnant and had an 18% increased risk of miscarriage. The highest risk was observed in women over 30 undergoing alkylating cytostatic treatment [120]. A matched cohort study showed a lower pregnancy rate in female cancer survivors compared to controls from the general population (13 vs. 22 %) [121]. In the largest studies, cancer patients were 35–38% less likely to become pregnant after cancer treatment compared to the general population, and each diagnosis was associated with a reduction in subsequent pregnancies [118,122]. While patients with malignant melanoma and thyroid cancer had a similar pregnancy rate compared to the control group, survivors of leukemia, cervical cancer, and breast cancer had the lowest subsequent pregnancy rate [118,121]. Among breast- and cervical cancer patients, the respective reduction in pregnancy rate was 60% and 66% compared to the general population [118]. To minimize pregnancy risks and complications, women with a history of previous cancer should be monitored at specialist maternity clinics. There is a recommendation to avoid pregnancy during, or in close connection to, a completed chemotherapy treatment. However, as cancer is a heterogeneous disease, advice for each patient should be individualized, as should advise on when it is appropriate to get pregnant. Two main parameters should be considered before counsel, the individual risk for relapse and the time until all gonadotoxic drugs have left the body [123].

## 5. Future Developments of Fertility Preservation

The field of FP has been under rapid development, and several treatment options for chemotherapy-induced infertility are still experimental, awaiting results from clinical trials, while other methods are less developed but have shown promise in early studies.

### 5.1. In Vitro Activation and Maturation of Primordial Follicles

In vitro culturing for 1–2 days of immature cumulus-oocyte complexes (COCs) from GV or metaphase I stage oocytes before fertilization is no longer regarded as experimental [7,77], and these methods have been used to achieve over a thousand live births since the first introduced in parallel with IVF in the seventies [124]. However, ex vivo maturation of immature follicles from cortical tissue is still under development [7] but might yet be a preferred future option to recover fertility when re-transplantation of ovarian tissue is not suitable. This could be the case when the risk of reintroducing malignancy is high, such as in patients with cancers with a high risk of ovarian metastasis or after systemic cancers such as leukemia [125]. In these cases, in vitro maturation of oocytes from ovarian tissue (OTO-IVM) shows great promise [126,127]. The method is still experimental since long-term safety studies are missing, but the first live births have been reported [128], and despite an observed lower oocyte maturation rate, the method remains a promising complement to OTC. 

Folliculogenesis is a complicated process, and so far, only one study has published results showing in vitro maturation of a human follicle from the primordial stage to maturity [129]. The latter stages of maturation have been thoroughly explored in vitro, and IVM from immature COCs is currently considered relatively safe, based on both epigenetic studies and clinical outcomes, despite the lowered fertility potential observed in IVM oocytes [130,131,132,133]. Activation of early-stage oocytes is less explored in a clinical setting. The phosphoinositide 3-kinase (PI3K)/Akt/forkhead box O3 (FOXO3) pathway regulates primordial follicle activation through a signaling cascade [134,135] and acts together with the Hippo pathway to promote follicle growth and survival and accelerate follicle recruitment [136,137]. Additionally, several other factors have been shown to both activate and inhibit folliculogenesis, among them AMH and FSH [138,139,140].

In situations where the woman has a low ovarian reserve at the time of FP, re-transplantation of cortical ovarian tissue is not expected to have a high success rate due to the massive follicle loss. Experimental studies have suggested that this obstacle could be partially overcome through in vitro activation (IVA) of the tissue, which would induce a synchronized burst of growing follicles to be harvested once they have matured in vivo [141]. There are multiple protocols for IVA aimed at overcoming growth arrest of the developing follicles, but the most common clinical approach involves ovarian fragmentation, with or without Act-stimulation (PTEN inhibitor and/or a PI3K stimulator). In conventional IVA the tissue is retransplanted after two days of in vitro stimulation and further follicle maturation is induced through gonadotropin stimulation paired with luteinizing hormone. This approach has led to healthy live births in patients with POI [142,143]. Opponents of IVA argue that in vitro stimulation limits the lifespan of the graft, which reduces long-term benefits, and that primordial follicle activation already occurs spontaneously after FP [144,145,146]. 

### 5.2. Protecting Ovarian Tissue In Vitro

While re-transplanting cryopreserved ovarian tissue is no longer regarded as experimental, the efficacy and long-term function of the ovarian graft is severely limited by the extensive follicle loss occurring through ischemic reperfusion injury following the transplantation. The PMFs are located in the outer part of the ovarian cortex, an area with relatively little vascularization [147], and it has been estimated that as much as two-thirds of the follicles are lost after grafting [148].

Treatments to protect the ovary from chemotherapy damage in vivo are highly sought after, and research on potential protectants has increased, many with good evidence of efficacy in experimental mouse models. Among the proposed protective agents are Sphingosine-1-phosphate, curcumin, capsaicin, and stem cells [16,149,150,151]. The promising results increase expectations for the future development of clinically effective treatments. The protectants examined to date have shown a potential to protect against all ovarian damage pathways except stromal tissue damage, and most act by blocking or downregulating the activated pathways, but some are also designed to directly reduce drug delivery to the ovary [19].

### 5.3. Stem Cells

The established truth of a non-generative pool of primordial follicles limiting the female reproductive potential was challenged by the apparent discovery of ovarian stem cells in adult mammals, including humans [152,153]. While ovarian germline stem cells might have great reproductive potential, studies in humans are limited, both because of ethical considerations and due to the scarcity of the cells. A study on mice showed that the incidence of ovarian stem cells decrease from 2% in neonates to only 0.05% in adult mice [154]. Other studies indicate that they do not exist at all, at least not in humans [155,156]. Despite the controversy, research is ongoing, and a recent study on post-menopausal women showed the possibility of identifying stem cells capable of maturating oocytes through advanced cell sorting [157]. The proposed germline cells’ capability to mature in vivo has been confirmed in several studies and supports functionality in adult mammalian ovaries through a capability to differentiate into oocyte-like cells and ultimately mature oocytes [158,159].

Several studies have also examined the use of adipose tissue-derived stem cells (ASC) in a mouse model, and the results, while inconclusive, indicate a positive effect on angiogenesis, follicle maturation, oxygenation, and apoptosis [160,161,162,163]. Stem cells have also been used in IVM, where the addition of human mesenchymal stem cells (MSC) from both umbilical cord and menstSSrual blood to follicle culture has increased follicular growth, decreased apoptosis, and improved survival [160,164].

## 6. Summary

Thanks to improved cancer treatments, long-term survival rates have increased. A growing patient group needs further developments in fertility preservation methods, and for most patients, an early referral for fertility counseling to a specialist in reproductive medicine is critical. Fertility preservation is a relatively novel field, and the long-term safety and efficacy of the methods are under continuous reporting. There is a need for large data to evaluate such procedures, and the creation of international registers should be encouraged. Clinical obstacles remain, but the treatment of the physiological aspects of infertility is improving at a reassuringly steady pace. While some women do seek help or get counseling, sexual dysfunction and the trauma linked to fear of recurrence and fear of infertility affect the larger patient group and often go untreated. As more and more young women will be cancer survivors, it is also important to address the less acute parameters limiting their chances to start or maintain a family.

Although embryo or oocyte cryopreservation is still the standard method for fertility preservation when a gonadotoxic treatment is planned, in vitro maturation and ovary tissue cryopreservation have recently been accepted in clinical practice. Resumed endocrine function after cortical ovarian tissue re-transplantation is expected, and the live birth rate is increasing, with over 300 children born to date. The use of protective agents to improve ovarian tissue cryopreservation, in vitro maturation of early follicles, and the use of stem cells to support fertility potential provide possible solutions to many of the practical difficulties in FP. Although human clinical trials are needed prior to implementation, new techniques combined with existing fertility preservation methods show promise for future improvement in treatment efficacy, especially for prepubertal girls and women in acute need of treatment.

## Figures and Tables

**Table 1:** Risk of gonadotoxicity associated with chemotherapy treatment [6,12,18,19].

High Risk	Medium Risk	Low Risk	Unknown Risk
Cyclophosphamide	Cisplatin ^1^	Methotrexate	Paclitaxel ^4^
Melphalan	Carboplatin ^1^	Bleomycin	Docetaxel ^4^
Busulfan	Adriamycin	Actinomycin D	Irinotecan
Procarbazine	Doxorubicin	Vincristine	Trastuzumab
Mustard gas derivatives		HL treatment ^2^	Imatinib
		5-flourouracil	Erlotinib
		Radioactive-iodine ^3^	Bevacizumab

^1^ Medium risk observed in treatments with low cumulative dose ^2^ Low risk observed in Hodgkin’s lymphoma treatments without alkylating agents ^3^ In treatments for thyroid cancer ^4^ In treatments for breast cancer.

## Data Availability

No new data was created.
